# Ophthalmic dispensing patterns in New Zealand: a ten-year review

**DOI:** 10.1038/s41433-026-04463-8

**Published:** 2026-05-05

**Authors:** Brandon Nunns, Vidit Singh, Jane Shi, James McKelvie

**Affiliations:** Department of Ophthalmology, University of Auckland, Auckland, New Zealand

**Keywords:** Education, Health services, Epidemiology, Epidemiology

## Abstract

**Background:**

Aimed to examine national dispensing patterns of ophthalmic medications in New Zealand over a ten-year period, focusing on demographic and regional variation.

**Methods:**

This nationwide population-level retrospective study analysed dispensing data for all Pharmaceutical Management Agency (PHARMAC)-subsidised ophthalmic medications between 1-January-2012 to 31-December-2021. Medications were classified into seven classes. Dispensing rates were compared by age, gender, ethnicity, and region.

**Results:**

10,941,081 dispensing events were recorded, increasing by 36% from 2012 to 2021. Chloramphenicol, Latanoprost, and Hypromellose with Dextran were the most dispensed agents. Anti-infective agents were mostly dispensed to the youngest and oldest age groups, dry eye and anti-inflammatory agents were dispensed mostly to females, and glaucoma medications were predominantly dispensed to older individuals. Age-adjusted rates demonstrated inequities for Māori, who accounted for 16.5% of the population but only 8% of dispensing events. Māori had consistently lower dispensing rates across most medication classes compared to European and the other ethnicity group, which included individuals identifying as any ethnicity other than Māori, European, or Pasifika, including Asian, Middle Eastern, Latin American, African, and all other ethnicities. Variation by geographic region was noted, with six of 20 regions exceeding the national mean dispensing rate of 246.42 per 1000 population per year.

**Conclusion:**

A substantial increase in dispensing of ophthalmic medications occurred in New Zealand over this 10-year period with significant variation by demographics. The inequity in dispensing rates for Māori is concerning and may contribute to healthcare inequity. These findings affirm the need for targeted strategies to improve equitable access to ophthalmic care and medications.

## Introduction

Ophthalmic medications form the mainstay of treatment for many ophthalmic conditions including glaucoma, dry eye disease, ocular infections, and ocular inflammatory disorders [1]. With an aging population in New Zealand, the prevalence of ophthalmic conditions, particularly glaucoma, is expected to increase [2]. This shifting demographic emphasises the importance of examining and understanding prescribing patterns to ensure optimal patient care and appropriate use of resources.

Existing research in New Zealand has concentrated on specific medication classes and conditions including glaucoma and microbial keratitis [3–5]. However, there has been limited research examining prescribing of all ophthalmic medications at a national level. In New Zealand, ophthalmic medications can be prescribed by a variety of healthcare professionals including doctors, nurse practitioners, and optometrists. Medications are publicly funded through the Pharmaceutical Management Agency (PHARMAC), which oversees the pharmaceutical schedule [6]. Medications listed on this schedule are subsidised and typically only require a small co-payment from patients, helping to improve accessibility [6]. Despite this funding model, there are many health inequities throughout New Zealand which are thought to exist due to systemic barriers and reduced access to care amongst other factors [7, 8]. These inequities also exist in ophthalmic outcomes, with previous research demonstrating a higher prevalence of advanced cataracts and sight-threatening diabetic eye disease in New Zealand Māori and Pasifika [7, 9, 10]. There is however, a lack of research focusing on identifying disparities in prescribing trends of all ophthalmic medications at a nationwide level.

This study aims to assess the dispensing patterns of ophthalmic medications across New Zealand over a ten-year period, focusing on differences in dispensing by geographic region and patient demographics including age, gender, and ethnicity.

## Methods

This is a nationwide population-level retrospective study examining the ophthalmic dispensing records from the Ministry of Health National Pharmacy dispensing database. Ethics approval was obtained from Auckland Health Research Ethics Committee (approval number AH24867), Waikato Hospital Research Officer, and Te Puna Oranga.

Data were extracted for all PHARMAC community-subsidised pharmaceutical eye drops, ointments, and injectable intravitreal agents dispensed on prescription to patients in the community across New Zealand between 1 January 2012 and 31 December 2021. Data provided from the database included medication names, de-identified encrypted National Health Index numbers, patient age, gender, ethnicity, date of dispensing, and geographic region of domicile. Each instance a medication is dispensed is recorded as a distinct event within the database and is referred to as a dispensing event throughout the study. Medication dispensing data were grouped into the following medication classes: anti-infective, anti-inflammatory, dry eye, glaucoma, anti-vascular endothelial growth factor (VEGF), mydriatic and cycloplegic, and other.

Population counts were obtained from census data from Statistics New Zealand [11]. The 2013 New Zealand census data were used to calculate dispensing rates between 2012 and 2017 [11]. The 2018 New Zealand census data were used to calculate dispensing rates between 2018 and 2021 [11]. Dispensing data for each medication class were standardised per 1000 population per year and used to calculate the mean annual dispensing rate per 1000 population across the study period. These rates were further analysed by geographic region of domicile and patient demographics including age, gender, and prioritised ethnicity, in which people of multiple ethnicities were classified as a single ethnicity in the order Māori, Pasifika, European, and Other. To account for differences in age-structure, ethnicity-specific dispensing rates were age-adjusted using New Zealand Māori as the standard population [11]. The Other ethnicity group includes all individuals who identified with an ethnicity not classified as Māori, Pasifika, or European. This group was predominantly composed of individuals of Asian ethnicity (85.8%), followed by those identifying as Middle Eastern, Latin American or African (7.5%), with the remainder consisting of smaller ethnic groups classified under the ‘other’ ethnicity group by Statistics New Zealand classifications [11]. Geographic areas were separated into 20 geographic regions of domicile in the study as listed under health regions by Statistics New Zealand [11]. This included Auckland, Bay of Plenty, Canterbury, Capital and Coast, Counties Manukau, Hawke’s Bay, Hutt Valley, Lakes, Midcentral, Nelson-Marlborough, Northland, South Canterbury, Southern, Tairawhiti, Waikato, Wairarapa, Waitemata, West Coast, and Whanganui.

Analysis was performed using R Statistical Software Package [12]. Records without age or ethnicity data were retained for calculations of overall dispensing events but were excluded from calculations of age-specific rates, age-adjusted ethnicity rates, and total dispensing events by ethnicity. Statistical analyses for comparison of dispensing rates utilised Welch’s *t* tests and one-way ANOVA. Post hoc Tukey’s HSD was used when appropriate. A *p*-value < 0.05 was regarded as significant.

## Results

**Fig. 1:**
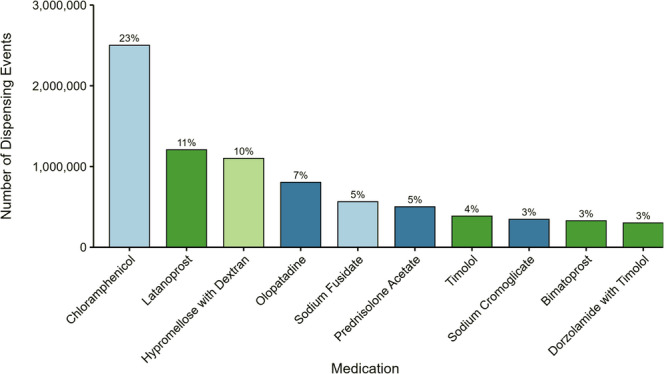
Total number of dispensing events over the study duration for the ten most dispensed ophthalmic medications. Each bar represents the total number of dispensing events of a single medication across the study period. Percentages of total dispensing events are displayed above each bar.

A total of 10,941,081 dispensing events were recorded over the ten-year study period. There were 946,046 dispensing events in 2012 and 1,291,047 dispensing events in 2021, with a mean number of yearly dispensing events of 1,094,108. The five most commonly dispensed medications were Chloramphenicol *n* = 2,501,614 (23%), Latanoprost *n* = 1,207,176 (11%), Hypromellose with Dextran *n* = 1,100,219 (10%), Olopatadine *n* = 804,041 (7%), and Fusidic Acid *n* = 563,954 (5%). A summary of the ten most dispensed medications is displayed in Fig. 1.

### Age

**Fig. 2:**
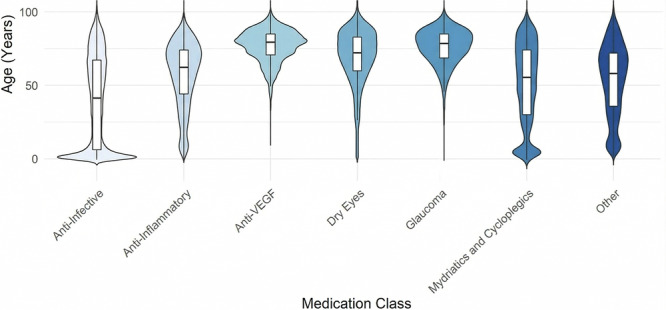
Age distribution at dispensing by ophthalmic medication class. Violin plots showing age distribution at dispensing for each medication class. Width represents data density. Medians and interquartile ranges are indicated by the embedded boxplots.

The age at time of dispensing varied for each medication class (Fig. 2). Anti-infective agents had the youngest mean age of dispensing at 39 (SD 31) years, followed by mydriatic agents at 51 (SD 28) years. Conversely, the oldest mean age was seen for anti-VEGF agents at 77 (SD 11) years and glaucoma agents at 76 (SD 13) years.

### Gender

There were 6,089,056 (55.65%) dispensing events for female patients compared to 4,698,195 (42.94%) for male patients (with the female and male population size ranging from 2,178,030 to 2,380,197, and 2,064,018 to 2,319,558, respectively, across the study). The remaining 153,830 (1.41%) dispensing events were to people of other and unknown gender. The rate of dispensing events for anti-inflammatory, dry eye, and mydriatic agents was significantly different between genders (*P* < 0.001, Table 1).

**Table 1:** Total dispensing events and mean rate of dispensing events per 1000 population per year by medication type and gender.

Medication class	Gender	Total dispensing events	Mean dispensing rate per 1000^a^	SD^a^	*P*-value^b^
Anti-Infective	Female	1,669,109	74.23	9.70	0.37
	Male	1,509,369	70.14	10.01	
Anti-Inflammatory	Female	1,071,584	47.41	1.64	<0.001
	Male	785,620	36.21	1.94	
Dry Eye	Female	1,014,780	44.53	11.27	<0.001
	Male	516,808	23.55	6.77	
Glaucoma	Female	1,581,678	69.86	8.16	0.25
	Male	1,417,905	65.14	9.68	
Anti-VEGF	Female	21,023	2.21	1.40	0.65
	Male	16,441	1.77	1.13	
Mydriatic & Cycloplegic	Female	37,447	1.65	0.17	<0.001
	Male	47,764	2.20	0.20	

^a^Mean and standard deviation of dispensing rate per 1000 population.

^b^*P*-value for difference in means by gender calculated by Welch’s *t* test.

### Ethnicity

Dispensing patterns also varied across ethnicity. The total number of dispensing events by ethnicity was 866,503 for Māori, 518,419 for Pasifika, 7,696,541 for European, and 1,708,910 for the other ethnicity group (with each ethnicity comprising 14.88–16.46%, 6.15–6.58%, 60.92–65.73%, and 13.24–16.03% of the population across the study period, respectively). Table 2 displays the rate of dispensing events by medication class and ethnicity and demonstrates significant differences in dispensing rates between ethnicities for all medication classes except for anti-VEGF agents (*P* < 0.01).

**Table 2:** Total dispensing events and age-adjusted mean rate of dispensing events per 1000 population per year by medication type and ethnicity.

Medication class	Ethnicity	Total Dispensing events	% of dispensing events	Mean % of population	Mean dispensing rate^a^	SD^a^	*P*-Value^b^
Anti-infective	Māori	448,390	14.10	15.67	69.01	15.97	0.009
	Pasifika	226,950	7.14	6.37	86.91	17.38	
	European	2,055,184	64.64	63.32	73.53	13.83	
	Other	448,893	14.12	14.63	92.61	18.58	
Anti-inflammatory	Māori	161,413	8.69	15.67	24.43	2.11	<0.001
	Pasifika	112,060	6.03	6.37	40.76	4.01	
	European	1,237,582	66.62	63.32	27.24	0.81	
	Other	346,716	18.66	14.63	52.45	5.88	
Dry eye	Māori	93,031	6.07	15.67	13.68	3.19	<0.001
	Pasifika	72,337	4.72	6.37	25.43	7.19	
	European	1,019,055	66.51	63.32	16.36	4.08	
	Other	347,645	22.69	14.63	46.45	14.70	
Glaucoma	Māori	56,963	1.90	15.67	8.45	1.39	<0.001
	Pasifika	40,990	1.37	6.37	14.50	3.08	
	European	2,630,225	87.66	63.32	34.68	4.71	
	Other	272,155	9.07	14.63	35.87	6.91	
Anti-VEGF	Māori	981	2.62	15.67	0.32	0.21	0.39
	Pasifika	1044	2.78	6.37	0.82	0.70	
	European	31,849	84.93	63.32	0.97	0.59	
	Other	3627	9.67	14.63	1.03	0.80	
Mydriatic & cycloplegic	Māori	11,093	13.01	15.67	1.67	0.21	<0.001
	Pasifika	5695	6.68	6.37	2.06	0.27	
	European	58,647	68.81	63.32	1.64	0.16	
	Other	9798	11.50	14.63	1.62	0.24	

Mean percentage of population calculated using the 2013 and 2018 census data.

^a^Age-adjusted mean and standard deviation of dispensing rate per 1000 population.

^b^*P*-value for difference in means by ethnicity calculated by one-way ANOVA test.

Significance testing with post hoc Tukey tests demonstrated significant differences in the rate of dispensing events between specific ethnic groups. Anti-infective agents were dispensed more frequently to people in the other ethnicity group compared to Māori (*P* = 0.01). Anti-inflammatory and dry eye agents were also dispensed more frequently to people in the other ethnicity group compared to the remaining ethnic groups (*P* < 0.001). Additionally, Pasifika had a higher rate of dispensing events for anti-inflammatory agents compared to Māori and European (*P* < 0.001) and for dry eye agents compared to Māori (*P* = 0.02).

Higher dispensing rates of glaucoma medications were observed for the European and other ethnicity groups compared to Māori and Pasifika (*P* < 0.001). Additionally, Pasifika had a higher rate of dispensing events for glaucoma medications compared to Māori (*P* = 0.02). The only significant difference in dispensing rates for mydriatic agents was a higher rate for Pasifika compared to all remaining ethnic groups (*P* < 0.01).

### Geographic region of domicile

**Fig. 3:**
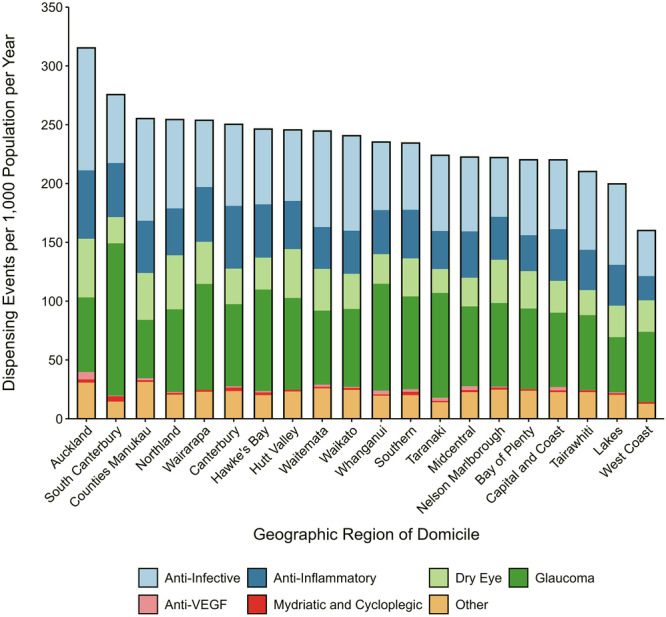
Mean annual dispensing rates per 1000 population by geographic region of domicile. Axes denote region (x-axis) and dispensing rate per 1000 population per year (y-axis). Each colour represents a distinct ophthalmic medication class as displayed.

The mean rate of dispensing events across all geographic regions of domicile was 246.42 per 1000 population per year. There were six regions with dispensing rates higher than the national average, including Auckland, Canterbury, Counties Manukau, Northland, South Canterbury, and Wairarapa. Variations in dispensing rate of each medication class by geographic region is displayed in Fig. 3.

## Discussion

The current study provides an analysis of dispensing patterns of ophthalmic medications in New Zealand over a ten-year period. It also identified differences in dispensing between different ages, genders, ethnicities, and geographic regions.

Across the study period, there was an increase in the number of dispensing events by 36% per year from 946,046 in 2012 to 1,291,047 in 2021, which is consistent with international trends demonstrating an increase in the utilisation of ophthalmic medications [13]. The observation nature of the current study cannot confirm a causal relationship, but the increase in New Zealand population size by 15% over the study period is likely to explain at least part of this increase [11]. Additionally, there was a 34% increase in the number of individuals aged 65 years or greater across the study period which is another likely contributing factor given those aged 65 years or older had the highest dispensing rates of ophthalmic medications in our study [11]. Another possible explanation is the increased availability of certain medications across the study such as the anti-VEGF agent Aflibercept which was first listed on the pharmaceutical schedule in 2018 [14].

The most commonly dispensed medication overall was Chloramphenicol, which is consistent with previously published research on antimicrobial dispensing in New Zealand [4]. Chloramphenicol is an antibiotic often used in the empiric treatment of conjunctivitis, prophylaxis following intraocular surgery, and as prophylaxis following wound closure for non-ophthalmic conditions [4, 15, 16]. Considering there are about 36,000 cataract surgeries each year, widespread post-operative use of Chloramphenicol would account for ~360,000 dispensing events across the study period [17]. However, previous research has shown no difference in the rates of endophthalmitis after cataract surgery with the use of Chloramphenicol, suggesting that a review of prescribing practices could be helpful to reduce unnecessary expenditure and development of antimicrobial resistance [18]. Another important consideration is that Chloramphenicol can be dispensed by Pharmacists without a prescription. These dispensing events are not included in our dataset and therefore the actual use of Chloramphenicol in the community is likely underestimated in our study. Similarly, the high dispensing rate of anti-inflammatory agents to older adults may in part be due to their use following cataract surgery [19] In contrast to the high use of Chloramphenicol, Ciprofloxacin was dispensed at much lower frequency, likely because it requires the prescribing health professional to specifically name an ‘endorsed condition’ such as microbial keratitis or refractory severe bacterial conjunctivitis on the prescription, [4, 5, 20]. This restriction is supported by increasing concerns regarding fluoroquinolone resistance, which has been observed in a recent New Zealand study examining resistance patterns of bacteria from corneal scrapes [21]. Our study found the dispensing of anti-infective agents was highest at the extremes of age. This finding may in part be explained by the higher rates of infective conjunctivitis in younger individuals, and the higher rates of ocular surgery in older adults in which Chloramphenicol may be used as a prophylactic agent [4, 15, 22]. Additionally, this study found that dispensing rates of anti-infective agents were highest in the Auckland and Counties Manukau regions, which also have the youngest median age of all regions and is consistent with the finding of higher dispensing rates of anti-infectives to younger individuals [11].

The second most commonly dispensed agent was Latanoprost, which is an anti-glaucoma agent [1]. Other anti-glaucoma agents included Timolol, Bimatoprost, Brinzolamide, Brimonidine, Travoprost, Pilocarpine, Dorzolamide, Betaxolol, and combination drops containing two anti-glaucoma agents [1]. The finding that Latanoprost was the most commonly dispensed anti-glaucoma agent is consistent with guidelines that suggest its use as an initial agent in the medical management of glaucoma [23]. Although other medications can also be used as an initial agent in the management of glaucoma, prostaglandin analogues are often used first because they are usually well tolerated, only require a single dose per day and have a relatively large effect on reducing intraocular pressure compared with other agents [23, 24]. We found that dispensing of glaucoma agents was highest for older individuals, which is consistent with the higher reported prevalence of glaucoma in older adults [23, 25]. There was also significant regional variation in the dispensing rate of glaucoma agents with South Canterbury having the highest rate of dispensing events, far in excess of other regions. Considering South Canterbury had an older median age, and a higher percentage of adults aged more than 85 years (2.55%) compared to all of New Zealand (1.82%), this is also in keeping with the higher prevalence of glaucoma in older adults [11, 23, 25]. Identifying the reasons for this finding was outside the scope of the study, however, differences in access to care and availability of ophthalmologists across regions may in part be responsible for this observation, along with the noted variations in age structure between regions.

It is also important to consider regulatory context when interpreting glaucoma dispensing trends. The Medicines Amendment Act 2013 was enacted in 2014 and classified optometrists within New Zealand as authorised prescribers, allowing them to prescribe anti-glaucoma agents that were previously restricted [26]. This legislative change shifted the prescribing landscape and may have contributed to the increase in glaucoma medication dispensing from 2014 onwards [26]. Analysis of dispensing at the prescriber-level is being explored in a companion study using prescriber-linked data. Regulatory changes to the availability of medications may have also influenced the dispensing rates of medications. Lattim, a combination eyedrop with Latanoprost and Timolol, is one such medicine that was added to the New Zealand Pharmaceutical Schedule as a publicly funded medicine in 2021 [27]. The availability of Lattim may have affected Latanoprost dispensing in the final year of the study, but a specific sub-analysis was not performed due to a limited availability of Lattim during the study period [27]. However, this change highlights the evolving prescribing landscape for glaucoma management in New Zealand [27]. A companion study is currently underway to examine the dispensing trends of individual glaucoma medications.

Variations in dispensing rates were also present by ethnicity with European individuals having higher dispensing rates of glaucoma agents compared to Māori and Pasifika. This finding is consistent with the reported significantly higher prevalence of glaucoma in European patients compared to Māori and Pasifika [25]. Other proposed explanations for this finding include genetic susceptibility, systemic barriers to accessing care, and the younger age structure of Māori although we have age-adjusted our results to account for this [25]. We also found a high rate of dispensing for glaucoma medications for the other ethnicity group compared to Māori and Pasifika, which may be because of the Asian and Indian ethnic groups within the other ethnicity category, as these ethnic groups have also been found to have a higher prevalence of glaucoma [2, 25].

The third most commonly dispensed agent was Hypromellose and Dextran, which is used to treat dry eye disease [28]. There were many other agents used for dry eye at reducing frequencies including Paraffin and Wool Fat, Polyvinyl Alcohol, Hyaluronic Acid, Retinol Palmitate, Macrogol-400 and Propylene Glycol, Hypromellose, and Carbomer. Our study found that the dispensing of medications for dry eye was skewed to older individuals, females compared to males, and those in the other ethnicity group compared to Māori, Pasifika, and European. These are expected findings since dry eye disease is known to be more common in older adults and in females, and at least in part explains the finding that there were more overall dispensing events to females (55.65%) compared to males (42.94%) [29]. Dry eye disease is also known to be more common in people of East Asian ethnicity, which may be responsible for the finding of higher dispensing rates in the other ethnicity group [29]. Anti-inflammatory agents were also dispensed at higher rates to females compared to males, which is consistent with research demonstrating a higher prevalence of ocular inflammatory disorders in females [30]. In contrast, males had a slightly higher dispensing rate of mydriatic agents, however the difference was small. Similarly, the difference in dispensing of mydriatic agents by age was small and may not be clinically important.

Our study also looked at the dispensing rates of anti-VEGF agents, which are used in the management of several ophthalmic conditions including age-related macular degeneration (AMD), diabetic macular oedema, and retinal vein occlusions [1]. We found the dispensing of anti-VEGF agents was skewed to older adults, which is expected given the prevalence of conditions treated with these agents, such as AMD, is also higher in older adults [31]. However, relative to the use of anti-VEGF agents in clinical practice, we report a low rate of dispensing. This is likely because Bevacizumab is not included within the dataset as it is supplied directly to hospitals and eye clinics rather than being dispensed to individual patients. The consequence of this is that the only anti-VEGF agent recorded in our study was Aflibercept in which eligibility criteria restrict funding to neovascular AMD, other causes of choroidal neovascular membranes, polypoidal choroidal vasculopathy, and diabetic macular oedema, only after a poor response to Bevacizumab, and in eyes with no structural damage to the fovea [32]. The result of this is that our study is likely to highly underestimate the usage of anti-VEGF agents and would underestimate the prevalence of diseases treated with them if used as a marker of prevalence. It also limits the potential of this study to detect inequities in the dispensing of these agents.

Our study identified several differences in dispensing across ethnicity, particularly for Māori whom accounted for 16.5% of the population but only 8% of dispensing events [11]. We have already explored reasons for the low dispensing rate of glaucoma medications in Māori, but this trend was also observed for anti-infective, anti-inflammatory, and dry eye agents. The prevalence of intraocular inflammatory disorders including uveitis is low in Māori and the prevalence of dry eye disease is not well known [7]. However, lower dispensing rates of anti-infective agents to Māori is in contrast to reported higher rates of ocular infections, including herpes simplex keratitis and certain forms of neonatal conjunctivitis [7]. The data from this study cannot determine the specific reasons for this inequity, but systemic barriers that disproportionately affect Māori are likely to have a role as seen in several other aspects of healthcare [7]. Additionally, differences in the social determinants of health between ethnic groups may also contribute to inequity [33]. A high proportion of Māori live in rural areas without a major hospital, which may disproportionately limit access to secondary care and specialist services for Māori [33]. The number of pharmacies in rural areas has also declined over several decades, further restricting access to ophthalmic medications in these areas [34]. There is also a higher proportion of Māori living in areas of the highest deprivation, which may further limit access to ophthalmic care and medications, and may contribute to the inequity in dispensing events seen in this study [33]. Further research should focus on strategies to address these barriers and inequities.

There are limitations to this study. Firstly, our study does not capture all ophthalmic medications being used in the community as the dataset does not include non-subsidised medications or medications dispensed by pharmacies without a prescription. The dataset also does not include practitioner and wholesale supply orders. Since some of these ophthalmic medications are supplied to and used primarily by hospitals, including anti-VEGF agents, it is not possible to use dispensing records to make any inference about disease prevalence or clinical practice. Additionally, the use of dispensing records underestimates total prescribing as medications that were prescribed but not dispensed were not captured. This underestimation in prescribing may be more pronounced in low socioeconomic populations due to difficulties in affording the co-payment for medications. Additionally, 1.38% of dispensing events recorded in the database had no associated age or ethnicity information recorded and were excluded from calculations involving age-adjustments, however these numbers were small. Overall, these limitations decrease the ability to make definitive conclusions regarding the appropriateness of prescribing practices, however it does give a real-world effect of clinical practice.

The results of this study are relevant to several areas of clinical practice. Firstly, the increased prescribing of ophthalmic medications across this study is an important reminder for clinicians to review their current prescribing practices. This is particularly important for the increasing use of anti-infective ophthalmic medications, which had the highest number of dispensing events in our study. There have been increasing concerns worldwide about antimicrobial resistance and reviewing prescribing practices to ensure good antimicrobial stewardship is one aspect to help address these concerns [35]. Perhaps most importantly, our study highlights inequities in dispensing of ophthalmic medications between ethnicities. Reduced dispensing of ophthalmic medications to particular ethnicities, as seen primarily for Māori in this study, may correspond to higher rates of preventable vision loss due to undertreated ocular conditions including glaucoma, and ocular infections. When considering local and national policy, emphasis should be placed on strategies to improve access and provide equitable care to avoid inequities in ophthalmic outcomes between ethnicities.

## Conclusion

This study analysed dispensing patterns of ophthalmic medications across New Zealand over a ten-year period. Overall, an increase in dispensing events across the study period was noted, consistent with international findings and an increasing and ageing population. We report significant variation in dispensing rates of different medication classes by age, gender, ethnicity, and geographic region of domicile. We observed, several inequities, particularly for Māori. These disparities should be the focus of future research to ensure equitable care for Māori in ophthalmic conditions and prescribing.

### What was known before:

- Existing ophthalmic medication class use: Existing research on ophthalmic medication use in New Zealand was focused on specific medication classes including glaucoma and microbial keratitis.
- Health inequities: Systemic health inequities are known to exist in New Zealand, with previous studies identifying higher prevalence of conditions such as advanced cataracts and sight threatening diabetic eye disease among Māori.

### What this study adds:

- Comprehensive use of ophthalmic medications. This study provides a comprehensive nationwide analysis of subsidised ophthalmic medication use in New Zealand.
- Identification of inequity of dispensing patterns across ethnicity. This study identifies significant disparities in dispensing of several ophthalmic medication classes for Māori.
- Establishes trends in medication use. This study identifies trends in medication dispensing by several demographic factors including age, gender, ethnicity, and geographic region. This highlights the importance of targeted strategies to improve equitable access to ophthalmic care.

## Data Availability

The data used in this study consists of national health data collected and held by the New Zealand Ministry of Health and is not openly available. Access to these data requires approval from the Ministry of Health. The authors obtained and used the data with the appropriate permissions.
